# TIM-1 acts a dual-attachment receptor for *Ebolavirus* by interacting directly with viral GP and the PS on the viral envelope

**DOI:** 10.1007/s13238-015-0220-y

**Published:** 2015-10-20

**Authors:** Shuai Yuan, Lei Cao, Hui Ling, Minghao Dang, Yao Sun, Xuyuan Zhang, Yutao Chen, Liguo Zhang, Dan Su, Xiangxi Wang, Zihe Rao

**Affiliations:** National Laboratory of Macromolecules, Institute of Biophysics, Chinese Academy of Science, Beijing, 100101 China; University of Chinese Academy of Sciences, Beijing, 100049 China; State Key Laboratory of Biotherapy/Collaborative Innovation Center of Biotherapy, West China Hospital, Sichuan University, Chengdu, 610041 China; College of Biotechnology, Tianjin University of Science and Technology, Tianjin, 300457 China

**Keywords:** *Ebolavirus*, viral entry, glycoprotein, receptor, interaction

## Abstract

*Ebolavirus* can cause hemorrhagic fever in humans with a mortality rate of 50%–90%. Currently, no approved vaccines and antiviral therapies are available. Human TIM1 is considered as an attachment factor for EBOV, enhancing viral infection through interaction with PS located on the viral envelope. However, reasons underlying the preferable usage of hTIM-1, but not other PS binding receptors by filovirus, remain unknown. We firstly demonstrated a direct interaction between hTIM-1 and EBOV GP *in vitro* and determined the crystal structures of the Ig V domains of hTIM-1 and hTIM-4. The binding region in hTIM-1 to EBOV GP was mapped by chimeras and mutation assays, which were designed based on structural analysis. Pseudovirion infection assays performed using hTIM-1 and its homologs as well as point mutants verified the location of the GP binding site and the importance of EBOV GP-hTIM-1 interaction in EBOV cellular entry.

**Electronic supplementary material:**

The online version of this article (doi:10.1007/s13238-015-0220-y) contains supplementary material, which is available to authorized users.

## INTRODUCTION

*Ebolavirus* (EBOV) and *Marburgvirus*, belonging to the *Filoviridae* family, cause hemorrhagic fever during the course of their infections in humans. Alarmingly, infections caused by these viruses have a high mortality rate of 50%–90% (Dolnik et al., 2008). In 2011, human T-cell immunoglobulin and mucin domain protein 1 (hTIM-1), previously implicated as a receptor for hepatitis A virus (Silberstein et al., 2003; Feigelstock et al., 1998; Wang et al., 2014), was reported as a receptor for EBOV and Marburgvirus (Kondratowicz et al., 2011). Recent studies indicate that hTIM-1, with some other but not all phosphatidylserine (PS) binding receptors, functions as a common attachment factor for a range of enveloped viruses, including filoviruses, flaviviruses, HIV, etc., through direct interaction with PS of the viral envelope (Li et al., 2014; Moller-Tank et al., 2013; Meertens et al., 2012; Jemielity et al., 2013). Interestingly, hTIM-1 can mediate uptake of viruses independent of the glycoproteins (Moller-Tank et al., 2013; Jemielity et al., 2013; Takada et al., 1997). However, recently hTIM-1 was shown to interact with NPC1, a fusion receptor for filovirus. This interaction was important for the entry of EBOV into host cell (Côté et al., 2011; Kuroda et al., 2015), thus raising an important question on whether hTIM-1 functions as an attachment factor or it is a bona fide receptor for EBOV.

TIM family proteins exhibit a classical type I membrane protein structure with an N-terminal immunoglobulin variable Ig-like (Ig V) domain, a heavily O-linked-glycosylated mucin-like domain (MLD) and a short C-terminal cytoplasmic tail (Freeman et al., 2010). Three human TIM proteins (hTIM-1, 3 and 4) and eight mouse TIM proteins (mTIM 1–8) have been identified so far. hTIM-1, 3 and 4 are considered direct orthologs of mTIM-1, 3 and 4, respectively (Kuchroo et al., 2003). More importantly, except for mTIM-2, the Ig V domains of all TIM proteins were predicted to contain a conserved PS binding site (Santiago et al., 2007). This is important in context of cellular entry of virus because PS is exposed on the membranes of various enveloped viruses and is known to play an important role in mediating viral entry (Mercer and Helenius, 2008; Soares et al., 2008). Intriguingly, PS receptor usage by different enveloped viruses for entry into host cells differs significantly, which might reflect distinct mechanisms involving additional interactions between viruses, PS receptors and other host factors (Moller-Tank et al., 2013; Jemielity et al., 2013). EBOV preferably utilizes hTIM-1, not other TIM proteins, to mediate the entry efficiently, which suggests that hTIM-1 probably has a direct or indirect interaction with EBOV glycoprotein (GP).

The GP of EBOV is composed of a trimer of disulfide-bonded GP1/GP2 heterodimers, which mediates receptor (s) binding, internalization, penetration and fusion with host-membranes (Takada et al., 1997; Wool-Lewis and Bates, 1998; Lee et al., 2008). GP1 contains the receptor binding domain (RBD), which is used to interact with its cellular receptor(s), while GP2 mediates virus/host membrane fusion events (Kuhn et al., 2006; Brindley et al., 2007). The GP is necessary and sufficient to mediate entry of EBOV or Ebola pseudovirion into plasma of host cells (Hunt et al., 2012).

Although methods used to study cellular entry of EBOV that rely on incorporation of Ebola GP onto surrogate viruses (HIV-1 pseudotypes) renders the study of EBOV entry easier, these methods do not exclude the possibility of a role for the PS located on the pseudovirion membrane in the process of viral entry into host cell. A structural view of the virus-receptor interaction or at least of the receptor would be more instructive about whether additional factors are involved in the attachment step. To clarify the role of hTIM-1 in cellular entry of EBOV, we first studied the interaction of hTIM-1 with GP of EBOV *in vitro*. We show that the Ig V domain of hTIM-1, but not other TIM family members, interacts directly with the GP and more specifically with the RBD of EBOV *in vitro*. Interestingly, the interaction was moderately inhibited by PS in a dose dependent manner. Furthermore, we determined the crystal structures of the Ig V domains of hTIM-1 and hTIM-4. Using the crystal structure as a guide we designed hTIM1-hTIM4 chimeras and studied their binding to EBOV GP to map the location of the GP binding site. Point mutants of hTIM-1 identified residues critical for GP-binding. Pseudovirion assays using point mutants of hTIM-1 confirmed the importance of the interaction interface in EBOV infection.

## RESULTS

### Preparation and characterization of Ig V domains of hTIM1, 3 and 4

**Figure 1:**
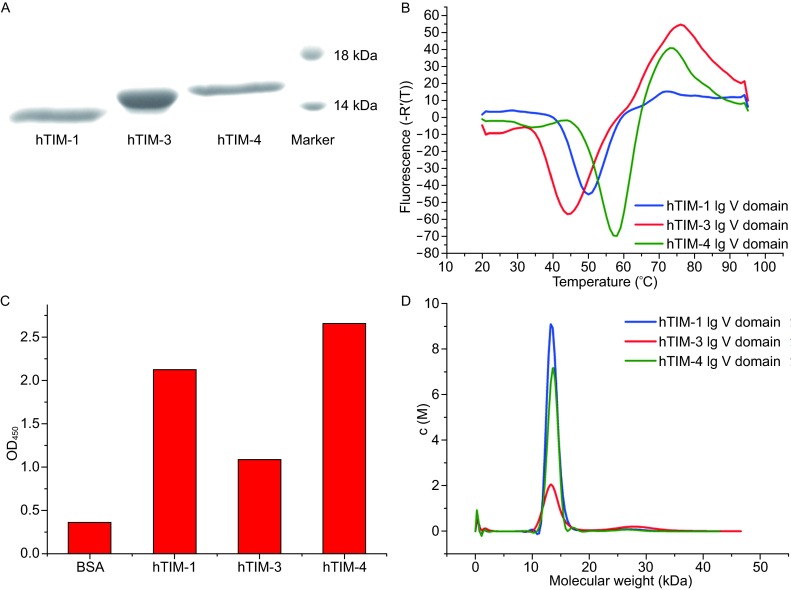
**Characterization of recombinant Ig V domains of hTIM-1, hTIM-3 and hTIM-4**. (A) SDS-PAGE analysis of Ig V domains of several human TIM proteins under reducing condition. (B) First derivatives of the fluorescence curves to measure the stability of Ig V domains of several human TIM proteins. Human TIM Ig V proteins were mixed with Sypro Orange dye and heated to indicated temperatures. Raw fluorescent signal increased as the dye bound to hydrophobic residues that were exposed from thermally destabilized proteins. (C) Binding of Ig V domains of several human TIM proteins to PS. Optical density (O.D.) was monitored at 450 nm to detect binding of human TIM proteins with Ig V domains to PS liposomes immobilized on plastic plates (See MATERIALS AND METHODS). (D) Analysis of oligomerization state of Ig V domains of several human TIM proteins in solution by sedimentation velocity experiments

Ig V domains of hTIM-1, 3 and 4 with a C-terminal 6× His tag were produced by using bacterial expression system as inclusion bodies. Ig V domains were successfully recovered from inclusion bodies by employing a de-naturation and refolding protocol. The proteins were further purified by Ni-NTA affinity chromatography and size exclusion chromatography (See “MATERIALS AND METHODS”). SDS-PAGE and thermal stability analysis as well as PS binding assays verified that the Ig V domains of hTIM-1, 3 and 4 represent functional units, with good purities and stabilities (Fig. 1A–C). Given the importance of oligomeric state in determination of the signaling mechanism of cell-surface receptors, the solution behaviors of the hTIM-1, 3 and 4 Ig V domains were examined. Sedimentation velocity experiments using an analytical ultra-centrifuge revealed that Ig V domains of hTIM-1, 3 and 4 existed as a monomer in solution (Fig. 1D).

### hTIM-1 Ig V is the only TIM family protein that interacts with Ebola GP

**Figure 2:**
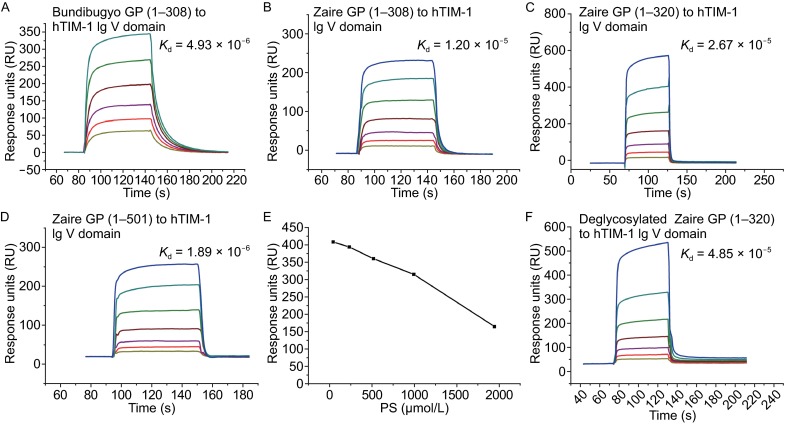
**Surface plasmon resonance assay for characterizing the specific binding between hTIM-1 and EBOV GP**. The profiles shown are as follows (A) Bundibugyo GP (1–308), (B) Zaire GP (1–308), (C) Zaire GP (1–320) and (D) Zaire GP (1–501) binding to hTIM-1 Ig V. (E) PS inhibited the binding between hTIM-1 and EBOV GP in a dose dependent manner. Response units were plotted against PS concentrations (See MATERIALS AND METHODS). (F) Binding of deglycosylated Zaire GP (1–320) to hTIM-1 Ig V. The binding affinities (*K*_d_) values were calculated using a steady-state affinity model produced with BIAcore 3000 analysis software (BIA evaluation Version 4.1)

To explore the potential interactions between TIM family proteins and EBOV GP *in vitro*, Ig V domains of mTIM1–4 (purchased from Sino Biological Inc.), hTIM-1, 3, 4 and recombinant GPs of Ebola (Zaire and Bundibugyo strain) were prepared (Fig. S1) (See MATERIALS AND METHODS). Results of binding assays performed using SPR showed that amongst the proteins tested only hTIM-1 Ig V could bind EBOV GP. To verify the specificity of the binding, 4 different constructs of EBOV GPs (Zaire GP 1–501, 1–320, 1–308 with a His tag and Bundibugyo GP 1–308 with a Fc tag) were tested. These constructs of GP presented a similarly high binding affinity of 4.9–26.7 μmol/L for hTIM-1 (Fig. 2A–D). Bundibugyo GP showed the strongest binding affinity for hTIM-1 Ig V with a dissociation constant (*K*_d_) of about 4.9 μmol/L, while GP and RBD from Zaire exhibited similar binding affinities for hTIM-1 Ig V (Fig. 2B–D). Interestingly the interaction between EBOV GP and hTIM-1 Ig V can be moderately inhibited by PS in a dose dependent manner. A 100-fold (mol/mol) excess PS in PBST buffer reduced binding efficiency of hTIM-1 to GP by ~25% (Fig. 2E), indicating the possibility that the binding sites of hTIM-1 to GP and PS overlap partially or fully. Deglycosylated RBD showed a ~50% reduction in binding affinity for hTIM-1, indicating that glycans on GP participate, but are not critical for the binding with hTIM-1 (Figs. 2F and S2).

### Structures of the Ig V domains of hTIM-1 and hTIM-4

**Figure 3:**
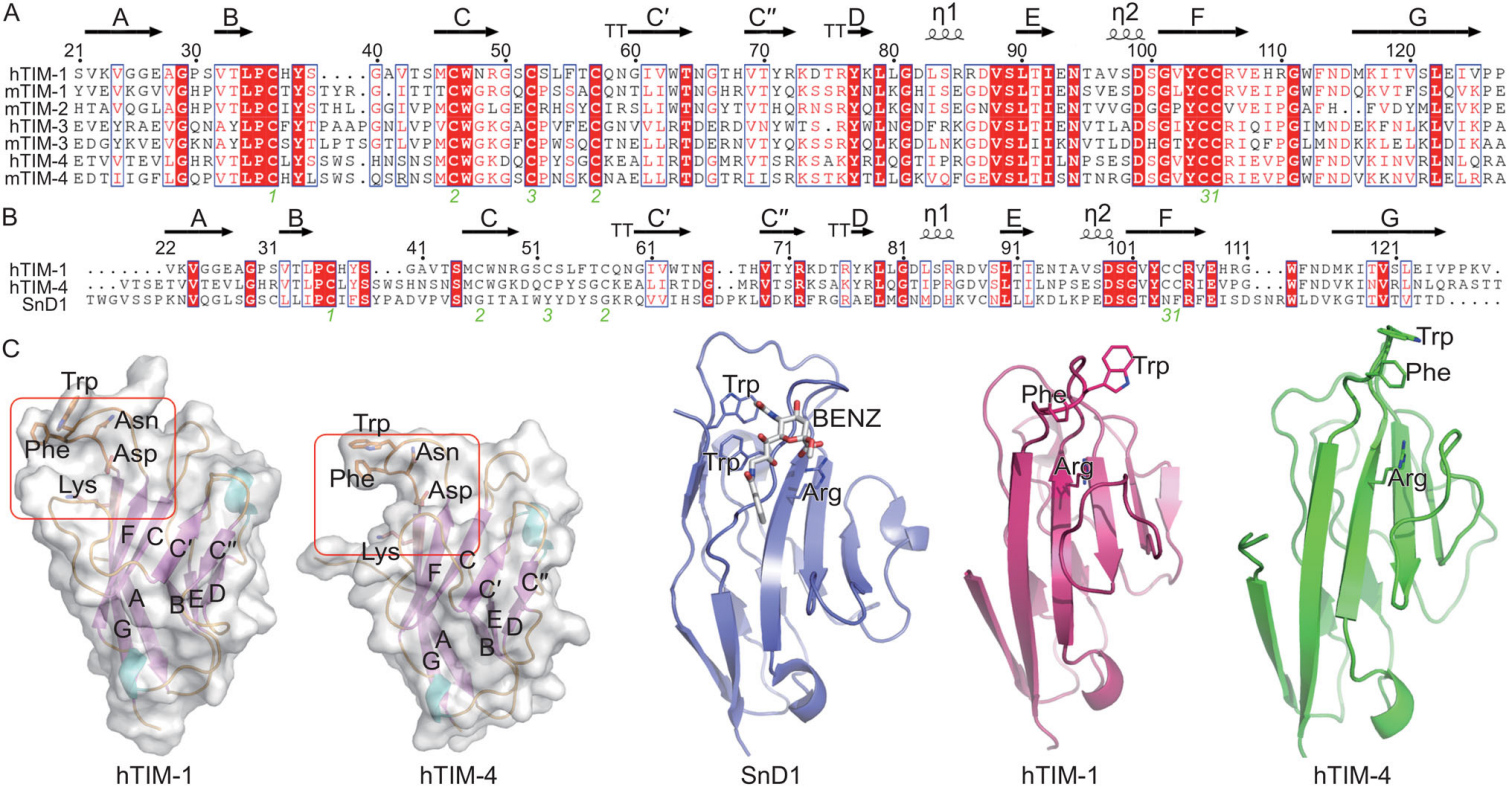
**Sequence alignment of hTIM-1 homologues and overall structures of Ig V domains of hTIM-1 and hTIM-4**. (A and B) Amino acid sequences of hTIM-1, hTIM-3, hTIM-4, mTIM 1-4 Ig V domains and hTIM-1, hTIM-4, sialecsialo adhesin (SnD1), respectively were aligned with the program Clustal W (Larkin et al., 2007). Cys residues and disulfide bonds are marked with green numbers. (C) Ribbon diagram of hTIM-1 and hTIM-4 Ig V domain structures. β strands are represented in magenta and labelled with capital characters, α helices and loops are colored in cyan and orange respectively. A cleft responsible for PS binding is marked with a red box, critical residues for ligand binding are shown as sticks. (D) Structural comparisons of hTIM-1 Ig V with hTIM-4 Ig V and SnD1 N-terminal Ig-like domain [PDB code: 1OD9] (Zaccai et al., 2003). Binding residues of SnD1 to sialated glycoconjugates and ligand (BENZ) are shown as sticks. Potential binding residues of hTIM-1 and hTIM-4 to sialated carbohydrates are also represented as sticks

We crystallized the Ig V domains of hTIM-1 and hTIM-4 and determined the structures at a resolution of 1.3 Å and 2.3 Å, respectively, using the molecular replacement method (See MATERIALS AND METHODS and Table S1). While hTIM-1 has only one molecule in the asymmetric unit, there are eight molecules of essentially the same structure present in the asymmetric unit of hTIM-4. The Ig V domains of hTIM-1 and hTIM-4, exhibit two antiparallel β sheets with the front and back faces formed by A, G, F, C, C′, C″ and B, E, D strands respectively (Fig. 3A and 3C). The overall structure closely mirrors the structure of mTIM-4 bound with PS. A hydrophobic cleft formed by CC’ and FG loops, located at an equivalent position in hTIM-1 and hTIM-4 structures represents the putative PS ligand binding site (Fig. 3C). Residues known to interact with PS, including WFND (Santiago et al., 2007) of the FG loop, are conserved among hTIM-1, mTIM-1, hTIM-4 and mTIM-4 (Fig. 3A). The amine group of the Ser residue of PS interacts with the Asp and the fatty acid moiety of PS is likely to be stabilized by the hydrophobic side chains of Trp and Phe (Fig. 3C). While mTIM-2 lacks the WFND motif (Fig. 3A) and therefore does not bind PS (Santiago et al., 2007), the aromatic residues, WF, of hTIM-3 and mTIM-3 are replaced with hydrophobic residues LM and IM, respectively. These substitutions lower the PS binding affinities of TIM-3 when compared to those of TIM-1 and TIM-4 (Figs. 1C and 3A). A striking structural observation that was confirmed by sequence analysis indicates that the Ig V domains of hTIM-1 and hTIM-4 share similarity with the N-terminal immunoglobulin-like domain of sialecsialo adhesin 1 (SnD1), hTIM-1 and hTIM-4 could be superimposed over the structure of SnD1 with an r.m.s. deviation of 2.3 Å and 2.2 Å, respectively, (Fig. 3B and 3D). This resemblance suggests that hTIM-1 and hTIM-4 Ig V domains might be able to bind carbohydrates using a siglec-like sialic acid binding motif. Three conserved residues (Trp 2, Arg 97 and Trp 106 in Siglec-1) together with a number of hydrophobic amino acids constitute sialic acid binding sites in all the siglecs (May et al., 1998). The putative PS binding clefts of hTIM-1 and hTIM-4 share the second and third (Arg and Trp) residue with siglecs. However the Trp is replaced by a Phe. Similar to other siglecs, hTIM-1 and hTIM-4 use hydrophobic residues of the CC’ loop to complete the binding site (Fig. 3B and 3D). Thus, our structural studies indicate that in addition to PS, hTIM-1 and hTIM-4 could possibly bind other ligands; in particular, carbohydrates.

### A unique groove adjacent to PS binding site in hTIM-1

**Figure 4:**
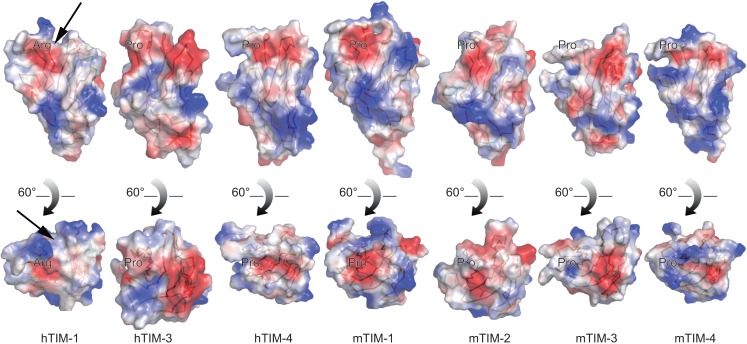
**Electrostatic surface features relevant to the cleft for substrate binding**. Distribution of charge on the surface of Ig V of TIM family proteins is shown [PDB code: 2OR8 (Santiago et al., 2007), 2OR7 (Santiago et al., 2007), 2OYP (Cao et al., 2007), 3BI9 (Santiago et al., 2007) and 4QYC (Huang et al., 2015)]. Blue color represents positive charge; red colour, negative. A unique groove formed by the BC and FG loops of hTIM-1 is indicated by a black arrow. Arg located on the FG loop in hTIM-1 and the equivalent residue (Pro) in other TIM family members is labelled

Despite sharing overall structural similarities with other Ig V domains of TIM family proteins, hTIM-1 Ig V domain exhibits unique features that may be essential for its distinct biological roles. Most notably, hTIM-1 possesses a significantly short BC loop that is the most variable structural feature amongst Ig V domains of the TIM family. In addition the residues of BC loop carry smaller side chains when compared to other TIM family proteins (Fig. 3A). This structural feature coupled with the movement of FG loop of hTIM-1 away from the BC loop, creates a unique groove that merges with the PS binding cleft (Fig. 4). Two residues (Arg 110 and Arg 86) in hTIM-1 (while the counterparts are Pro and Gly, respectively in other TIM family members) render the two walls of the groove positively charged (Fig. 4). To find out why only hTIM-1 but not other TIM proteins bound EBOV GP, we looked at structures of hTIM-1 homologues. In mTIM-1 structure, as the FG loop moves forward towards the BC loop, the FG and BC loops are connected by the Pro and Arg residues, forming a channel (Fig. 4). For hTIM-3, the longer strands of βC and βG approach to each other, so that the FG and BC loops are too close to form a groove, whilst, a fully negatively charged valley is formed by BC loop and C’C’’ loop (Fig. 4). On the contrary, the BC loop of mTIM-3 extends away from the FG by up to 11.3 Å, leading to a flat and broader groove with negatively charged decoration on one side (Fig. 4). As to hTIM-4 and mTIM-4, three extra residues from the BC loop fill in the gap between the FG and BC loop (Fig. 4). Thus, the surface characteristics; in particular, the putative ligand-binding groove of hTIM-1 are different from those of hTIM-1 homologues. This could possibly explain why only hTIM-1 and not other TIM proteins bind EBOV GP.

### Multiple sites contribute to the binding of hTIM-1 to Ebola GP

Given that hTIM-1 is the sole target among TIM family to interact with Ebola GP, at least one of three loops (the BC, CC’ and FG loops) are expected to participate in the interaction with Ebola GP. This inference is based on the structural analysis between hTIM-1 and other TIM family proteins (Fig. 4) where the loops are involved in formation of cavities for ligand binding. A series of chimeras of hTIM-1 that binds EBOV GP and hTIM-4 that does not bind EBOV GP, where corresponding structural elements were exchanged, were generated to map the region(s) that are important for Ebola GP binding (Fig. S3 and See MATERIALS AND METHODS). In the T14 (BC) mutant, for example, the BC loop of hTIM-1 was replaced with the corresponding region of hTIM-4. We hypothesized that the binding affinity of EBOV GP to hTIM-1 would be lowered by replacement of this important region with the corresponding region from hTIM-4 and vice versa. We produced these chimeric mutants using a similar strategy as that used for producing hTIM-1 and verified them as functional units by thermal stability assays (Fig. S4). The binding affinities of EBOV GP to these chimeras were determined by SPR. As expected, the T14 (BC-CC’-FG) chimera showed the most dramatic loss in binding activity to GP, whilst the T41 (BC-CC’-FG) chimera restored the binding activity to GP (Table 1 and Fig. S5). Remarkably, the T14 (FG) chimera exhibited only a slightly lower affinity for Ebola GP, while, both the T14 (BC) and T14 (CC’) chimeras decreased the binding affinity by 10 folds (Table 1 and Fig. S5). In agreement with hTIM-1 chimeras, the T41 (BC-CC’) and T41 (CC’-FG) chimeras gained some ability of binding to GP, but with a lower affinity of 73 and 208 μmol/L, respectively (Table 1 and Fig. S5). An interesting observation coming out of the studies using chimeras was that exchanging FG loops did not significantly reduce the binding affinity. This can be explained from the fact that except for Arg110, all other residues of FG loop from hTIM-1 and hTIM-4 are highly conserved. However, it should be noted that no significant change in binding affinity for FG loop chimeras does not necessarily mean that FG loop is not critical for Ebola GP binding. To address this issue, we constructed point mutants of residues from the FG loop of hTIM-1.

**Table 1:** Binding affinity measurements of hTIM-1 Ig V mutants to EBOV GP

Mutation	Location	Affinity
hTIM-1	–	2.67 × 10^−5^
R86A	DE loop	5.11 × 10^−5^
R106A	F strand	3.92 × 10^−4^
R110A	FG loop	4.95 × 10^−5^
W112A	FG loop	–
F113A	FG loop	–
N114A	FG loop	4.01 × 10^−4^
D115A	FG loop	8.65 × 10^−3^
K117A	G strand	1.96 × 10^−4^
R106A/K117A	–	–
T14 (BC)	–	–
T14 (CC’)	–	–
T14 (FG)	–	5.79 × 10^−5^
T41 (BC-CC’)	–	7.32 × 10^−5^
T41 (CC’-FG)	–	2.08 × 10^−4^
T14 (BC-CC’-FG)	–	–
T41 (BC-CC’-FG)	–	7.24 × 10^−5^

Binding affinity is assessed by SPR

“–” means no detectable binding signals

Single Ala substitutions of the 4 residues (W112/A112 or F113/A113 or N114/A114 or D115/A115) of the FG loop abolished the binding of hTIM-1 to Ebola GP (Table 1 and Fig. S5). The results are consistent with that in the case of the PS binding, all the four residues of WFND of the FG loop have been shown to be crucial for binding the ligand (Santiago et al., 2007). Two positively charged residues Arg 106 and Lys 117 in hTIM-1, which are conserved in the TIM family, are speculated to interact with the carboxylate group of the PS or sialic carbohydrate (Fig. 3C and 3D), while, these two residues were verified to play a key role in the interaction with Ebola GP as well. Single mutation of these two residues (R106/A106 or K117/A117) decreased binding of hTIM-1 to Ebola GP by ~90% and double mutations (R106K117/A106A117) lost completely the binding ability (Table 1 and Fig. S5). In addition, the single mutations R86/A86 and R110/A110, exhibiting 60% and 50% reduction, respectively, in the binding affinity of hTIM-1 to Ebola GP, confirmed the structural interpretation that the unique groove in hTIM-1 also participates in GP binding (Table 1 and Fig. S5). Based on the above results, we conclude that all the three loops, BC, CC’ and FG, of hTIM-1 together constitute the binding sites to Ebola GP, of which the residues Arg 106, Trp 112, Phe 113, Asn 114, Asp 115 and Lys 117 are critically important for Ebola GP binding, while, the residues Arg 86, Arg 110 have moderate effects on GP binding.

### hTIM-1 mediated Ebola pseudovirus entry integrates multiple factors

**Figure 5:**
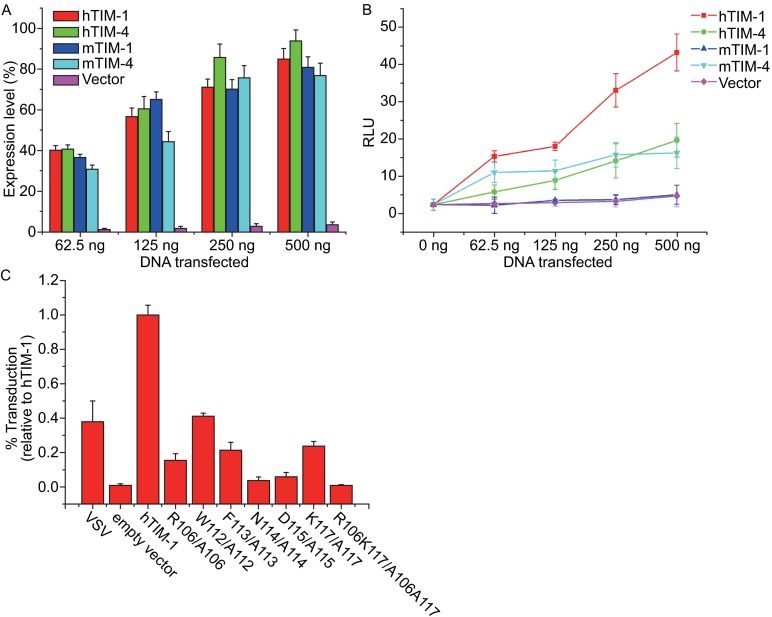
**hTIM-1 serves as a dual-attachment receptor for EBOV and identification of hTIM-1 Ig V residues that impact EBOV GP-dependent entry**. (A) Surface expression of hTIM-1, hTIM-4, mTIM-1 and mTIM-4 at 48 h following transfection. HEK 293T cells were transfected with increasing amounts of TIM expression plasmids or empty plasmids (62.5 ng, 125 ng, 250 ng and 500 ng), the expressions of TIM proteins were assessed by flow cytometry using anti-flag antibodies. (B) Compare the transduction efficiency of EBOV pseudovirus mediated by hTIM-1, hTIM-4, mTIM-1 and mTIM-4. At 48 h, transduce EBOV pseudovirions into HEK 293T cells, which were transfected with a growing amount of a hTIM-1 or hTIM-4 or mTIM-1 or mTIM-4 expression plasmid (62.5 ng, 125 ng, 250 ng and 500 ng). Luminescence signals are expressed as relative light units (RLU). (C) Identification of hTIM-1 Ig V residues that impact EBOV pseudovirion entry. Compare the transduction efficiency of EBOV pseudovirions into HEK 293T cells transfected by hTIM-1 mutant constructs to that with WT hTIM-1. Empty plasmid transfected HEK 293T cells were used as a negative control. Transduction of VSV pseudovirions into HEK 293T cells is a positive control. Results shown are the mean ± SEM of three independent experiments for panel (A–C)

Integration of viral GP into surrogate viruses (VSV or HIV pseudotypes) has allowed the EBOV entry to be studied in a biosafety level 2 setting. hTIM-1 was reported to promote some pseudoviruses entry by binding to the PS on the virion surfaces; in particular, those from the filovirus, flavivirus and alphavirus families (Jemielity et al., 2013). Although various enveloped viruses and pseudoviruses contain PS on their membrane (Mercer and Helenius, 2008), hTIM-1 is not able to promote infection of all pseudoviruses (Moller-Tank et al., 2013), which suggests that mechanisms of cellular entry differ for different viruses. To compare the ability of TIM family proteins to mediate EBOV pseudoviruses transduction systematically, we cloned hTIM-1, hTIM-4, mTIM-1 and mTIM-4 into pCMV3-SP vector with a flag tag, evaluated the surface expression of the 4 proteins in 293 T cells using flow cytometric assay with an anti-flag antibody (See MATERIALS AND METHODS). Expression levels of hTIM-1, hTIM-4, mTIM-1 and mTIM-4 on the cell surface were close by transient transfection of the indicated amounts of a TIM-expressing plasmid (Fig. 5A), but hTIM-1 enhanced EBOV pseudovirus entry significantly greater than hTIM-4 and mTIM-4 (>3-fold when transfected with 0.5 μg) (Fig. 5B). Surprisingly, expression of mTIM-1 did not enhance EBOV pseudovirus transduction. Together with the fact that hTIM-3 only enhanced EBOV entry very inefficiently (Kondratowicz et al., 2011; Moller-Tank et al., 2013), these indicate that the efficiency of viral entry via interaction of TIM proteins with PS of the viral membrane is low when compared to the one mediated by direct binding between hTIM-1 and GP. This argument is also supported by the fact that EBOV GP-bearing VP40-GFP VLPs appeared to be internalized much more efficiently than those lacking GP (Jemielity et al., 2013). Meanwhile, this argument is also consisted with lower binding affinity of hTIM1 to PS than to GP (Fig. 1E). Previously PS was reported to block the entry of EBOV into hTIM1-expressing 293T cells at the concentration of 10 μmol/L (Jemielity et al., 2013), however, the concentration of hTIM-1 molecules on the hTIM1-expressing 293T cell surface is 10–100 nmol/L based on the expression level analysis, it is not surprising that an approximate over 100-fold (mol/mol) excess PS can inhibit EBOV entry at the cellular level. EBOV entry mediated by hTIM-1 results from a dual-interaction of viral GP and PS with hTIM-1 receptor. Consistent with the results of *in vitro* binding assays performed using purified recombinant proteins, mutants of hTIM-1 (R106/A106, W112/A112, F113/A113, N114/A114, D115/A115, K117/A117 and R106K117/A106A117), with an equivalent expression level to that of the wild type, lower deficient in facilitating cellular entry of Ebola pseudovirus due to the inability of binding of viral PS or GP to the mutant hTIM-1 receptor (Fig. 5C).

## DISCUSSION

There is an increased appreciation that the PS receptors, such as TIM family proteins, play important roles in viral infection (Moller-Tank et al., 2013; Jemielity et al., 2013; Rennert, 2011). A number of lines of evidence indicate that hTIM-1 enhances infection of a variety of enveloped viruses by binding to virion-associated PS and does not require viral entry protein (Moller-Tank et al., 2013; Meertens et al., 2012; Jemielity et al., 2013), therefore hTIM-1 is generally described as an attachment factor. Contrary to this, preferable usage of hTIM-1, not other PS receptors by filoviruses and flaviviruses suggests hTIM-1 may not just function to enhance the attachment of the virion-associated PS to cell surface. Moreover, structural analysis reporting that the membrane of flaviviruses are occluded by tightly-arranged viral glycoproteins further weakens the argument that TIM-1 is an accessory protein that enhances PS-mediated viral uptake (Zhang et al., 2003a, 2003b). Lastly, hTIM-1 was recently shown to interact with NPC1 to facilitate EBOV GP-mediated membrane fusion (Kuroda et al., 2015) and direct interaction *in vitro* between hTIM-1 Ig V and NPC1 C-loop domain was also demonstrated by SPR (data not shown), suggesting that hTIM-1 might have additional roles and not function exclusively as an attachment factor for EBOV. In this context, our studies demonstrating a direct interaction between hTIM-1, but not other TIM family proteins, and EBOV GP *in vitro* by SPR confers a much bigger role on hTIM-1 in cellular entry of EBOV. We show that hTIM-1 can act as a dual-attachment receptor to recruit EBOV to cell surface by interacting directly with GP and the PS on the viral envelop. Based on our results of *in vitro* binding affinity studies and previously reported pseudovirion assays (Jemielity et al., 2013), the contribution of GP to mediating EBOV entry appears to be more significant than that by PS.

The structures of Ig V domains of hTIM-1 and hTIM-4 reveal presence of a conserved hydrophobic cavity used by the TIM proteins for binding to PS. The ligand binding site is characterized by a constrained size of the cavity and the requirement for both an acidic group and a hydrophobic group in the ligand (Fig. 3C). Structural comparisons and amino acids sequence analysis with the N-terminal Ig-like domain of the Siglec family suggest that sialated glycoconjugates with an acidic group at the head and a hydrophobic group at the tail might constitute new ligands for hTIM-1/hTIM-4 (Fig. 3B and 3D). Sialic acids are mainly terminal components of glycans on glycoproteins and these sialated carbohydrates are involved in numerous cellular recognition process, such as pathogens adhesion (Kelm and Schauer, 1997; Schauer and Kamerling, 1995; Angata and Varki, 2002). EBOV GP was reported to bear sialic acid residues at the termini of a small fraction of glycans (Powlesland et al., 2008). These observations coupled with the results that deglycosylated GP exhibited a ~50% reduction in binding affinity to hTIM-1 (Fig. 2F), allow us to propose that sialated carbohydrates might be additional ligands of hTIM-1/hTIM-4.

We compared the amino acid sequences and structures of hTIM-1 with other TIM family proteins, and designed a series of hTIM-1-hTIM-4 chimeras and mutants to map the regions of hTIM-1 that are important for EBOV GP binding. Three key loops (BC, CC’ and FG loops) of hTIM-1 participate in interaction with EBOV GP (Table 1). Amongst these, the BC and FG loops in hTIM-1 form an unique groove-like structure adjacent to the PS binding site, which is in-turn formed by FG and CC’ loops (Figs. 3C and 4). Additionally, we further identified a number of residues on these three loops, which are critical for the EBOV GP binding (Table 1). It is interesting to note that the binding site of PS to hTIM-1 is a subset or continuation of EBOV GP binding site on hTIM-1, which explains why hTIM-1 binds to GP with a higher affinity than hTIM-1 binds to PS.

A recent study has concluded that hTIM-1 is not only involved in filovirus attachment but it also participates in efficient membrane fusion through its interaction with NPC1 in late endosomes (Kuroda et al., 2015). Interestingly, we observed that the affinity between hTIM-1 and EBOV GP increases as the pH drops to that observed in endosomes (pH 7.5–5.7) (Fig. S6). It is possible that hTIM-1 acts as a bridge to facilitate the binding of NPC1 to GP, initializing conformational changes in GP to trigger membrane fusion.

In summary, we firstly identified the direct interaction between hTIM-1 and EBOV GP *in vitro* by SPR assay and solved the structures of the Ig V domains of hTIM-1 and hTIM-4 by crystallography, then mapped the regions in hTIM-1 that are important for EBOV GP binding. Inferences gained from results of our biophysical and biochemical studies have been verified by pseudovirion infection assays. Targeting the hTIM-1-EBOV GP interaction interface to block the binding of EBOV GP to hTIM-1 could be a promising strategy for stalling the attachment step of Ebola virus infection.

## MATERIALS AND METHODS

### Structure determination

Materials, such as cells and plasmids, and methods for protein purification, crystallization and the PS binding assay can be found in the Supplementary Materials. Diffraction data sets for hTIM-1 and hTIM-4 Ig V domains were collected at beam line BL17U and BL19U of the Shanghai synchrotron facility with the highest resolution being 1.3 Å and 2.3 Å, belonging to space groups of *P4*_*3*_*2*_*1*_*2* and *P2*_*1*_*2*_*1*_*2*_*1,*_ respectively. Datasets were processed and scaled using the HKL2000 package (Otwinowski and Minor, 1997). One and eight protein molecules in an asymmetric unit with a solvent content of 52% and 59% (corresponding to a Matthews coefficient V_M_= 2.55 and 2.96 Å^3^ Da^-1^ (Matthews, 1968)) were found from crystals of hTIM-1 and hTIM-4, respectively. The initial structure solutions of hTIM-1 and hTIM-4 were obtained by molecular replacement using the program Phaser v2.1 (McCoy et al., 2007) with the crystal structure of mTIM-1 (Protein Data Bank [PDB] entry: 2OR8 (Santiago et al., 2007)) and hTIM-1 respectively as a search template. Manual model building and refinement were performed using COOT (Emsley and Cowtan, 2004) and PHENIX (Adams et al., 2010). The r.m.s. deviations between the eight NCS-related subunits of hTIM-4 are less than 0.12 Å. Chain A from hTIM-4 was selected for following structural analysis. Structural figures were drawn with the program PyMOL (DeLano, 2002).

### Flow cytometric assay

For the surface expression of hTIM-1, hTIM-4, mTIM-1 and mTIM-4, the coding sequences which start from right behind the signal peptide and last to the end were cloned into the pCMV3-SP-N-FLAG vector. So that the proteins expressed on the cell surface all have a flag tag linking at the N terminal. The plasmids were transfected into TIM1 ^-^/^-^ 293T cells using lipofectamine2000 according to the manufacturer’s instructions (Invitrogen). The cells were collected 48 h after transfection. To measure the expression, cells were stained with PE anti-flag antibody and with PE rat Ig G2a antibody as negative control (Biolegend). 7-AAD was used to monitor the dead cells through binding to the nucleic acid. The data was analyzed using the Summit v4.3 software.

### Surface plasmon resonance assay

The BIAcore experiments were carried out at room temperature (25°C) using a BIAcore 3000 machine with CM5 chips. For all the measurements, a PBST buffer consisting of PBS, pH 7.4 and 0.005 % (*v*/*v*) Tween-20 was used. The Ebola GP proteins were immobilized on the chip at about 7000 response units. Gradient concentrations of human TIM1 and its mutations (0, 0.625, 1.25, 2.5, 5, 10, 20, 40 μmol/L) were then used to flow over the chip surface. The binding kinetics were analysed with the software BIAevaluation Version 4.1 using the steady state affinity. To study the effect of the ligand PS and pH on the binding of hTIM-1 to EBOV GP, the concentration of hTIM-1 was maintained constant (10 μmol/L). The concentration of PS was gradually increased (PS: 200 μmol/L, 500 μmol/L, 1 mmol/L, 2 mmol/L) in the binding buffer. Similarly, the buffer pH was gradually changed from 7.5 to 5.7 in steps of 0.3 units. The response values were recorded to analyze the changes.

### Thermofluor assay

Thermofluor experiments were performed with iCycleriQ Real Time PCR Detection System (Bio-Rad) instrument. SYPRO orange was used as fluorescent probe to monitor the denaturation of the proteins. 25 µL reactions were set up in a PCR plate, containing 0.1 mg/mL each protein, 5× Sypro Orange in pH ranging from 4.0 to 8.4 buffer solutions and ramped from 20–95°C with fluorescence recorded in triplicate at 1°C intervals.

### Pseudovirion

Pseudovirions bearing Ebola virus GP protein were produced in 293T cells transfected with PNL 4-3 vector containing Luciferase as a reporter and pcDNA3.1 vector, which contains GP, using lipofectamine2000 according to the manufacturer’s instructions. Pseudovirus-containing culture supernatant was harvested at 48 h post-transfection and stored at −80°C.

For infection, cells were plated on 96-well plates and incubated at 37°C with serially diluted pseudovirus-containing supernatant to yield proper level of infection. Supernatants were replaced with fresh medium after 3 h of incubation post infection by pseudovirus. Another 48 h is needed to allow for luciferase reporter to expression and infection levels were assessed by measuring bioluminescence signals.

### Analytical ultracentrifugation

Sedimentation velocity experiments were performed on a Beckman XL-I analytical ultracentrifuge at 20°C. Protein samples were diluted with lysis buffer to 400 μL at an A_280nm_ absorption of about 0.8. Samples were loaded into a conventional double-sector quartz cell and mounted in a Beckman four-hole An-60 Ti rotor. Data were collected at 60,000 rpm at a wavelength of 280 nm. Interference sedimentation coefficient distributions were calculated from the sedimentation velocity data using the SEDFIT software program (www.analyticalultracentrifugation.com).

## Electronic supplementary material

Supplementary material 1 (PDF 906 kb)
