# Supplementary material for: TIM-1 acts a dual-attachment receptor for Ebolavirus by interacting directly with viral GP and the PS on the viral envelope
Source: Protein Cell. 2015 Oct 20;6(11):814–24. doi: 10.1007/s13238-015-0220-y (PMC4624681; doi:10.1007/s13238-015-0220-y)
Supplement: Supplementary file 1 — Supplementary material 1 (PDF 906 kb) [file 13238_2015_220_MOESM1_ESM.pdf]

Supporting Information for

TIM-1 acts a dual-attachment receptor for *Ebolavirus* by interacting directly with viral GP and the PS on the viral envelope

This document includes:

SI text

Table. S1

Fig. S1--- Fig. S6

SI text

SI Materials and Methods

Protein purification and Crystallization

The Ig V domains of human TIM-1 (residues 22-129) and TIM-4 (residues 22-137) were cloned in pET-22b vector (Novagen) between Nde I and Xho I restriction sites. These proteins containing a C-terminal 6×His-tag were expressed as inclusion bodies in *E. coli* Bl21(DE3). Protein from inclusion bodies was solubilized by using a de-naturation and refolding protocol. The refolding buffer (50mM Hepes, 0.4M L-Arginine, 6.3mM GSH, 3.7mM GSSG, 2mM EDTA, pH 7.0) was used to refold both the Ig V domains of hTIM-1 and hTIM-4 which were denatured with the buffer of 20mM Tris, 6M Guanidine Hydrochloride, 2mM DTT, 10mM EDTA, pH 8.5. The denatured proteins were dialyzed against a 100-fold excess of refolding buffer. The re-folded proteins were purified further using Ni-NTA affinity chromatography. The Ig V domains of human TIM1 and TIM4 eluted in a single peak in the subsequent step of size exclusion chromatography performed using a Superdex G75 column. The Ig V domain of hTIM-3 (22-135) was purified in a similar way.

Zaire GP (1-320) was cloned to pfastbac-bee vector and Sf9 cells were transfected with recombinant bacmid. Recombinant GP was produced using Sf9 cells by infecting 1 lit of cells (2 x 10^6/ml confluence) with 10 ml of P3 viral stock that was obtained following manufacturer's instructions (Invitrogen). The medium containing the secreted protein was collected after 3–4 days of incubation. The cell culture medium of Sf9 cells was harvested and centrifuged for 30min at 4000g. The supernatant was filtered through a 0.22 μm filter and then dialysed against 10-fold excess of PBS (pH=7.4) and purified further by Ni-NTA affinity chromatography and size exclusion chromatography (superdex G200). Zaire GP (1-308 and 1-501) and

Bundibugyo GP (1-308) were purchased from Sino Biological Inc.

Crystals of the Ig V domain of human TIM-1 (4 mg/ml) grew in 1.5 M sodium nitrate, 0.1 M sodium acetate trihydrate, pH 4.6 after incubation at 20 ℃ for 1 day. Crystals of the Ig V domain of human TIM4 (8 mg/ml) were obtained from the condition of 20% (w/v) PEG 6000, 100 mM citric acid/sodium hydroxide, pH 5.0 after two days at 20 ℃.

ELISA assay

To ensure that the proteins were refolded correctly, we tested the ability of the refolded proteins to bind its ligand PS using ELISA-based assays. The PS used to test the activity of TIM proteins has a 6:0 tail (Avanti). Briefly, 96-well plates were coated with 100 μl $well^{-1}$ of PS (50μg ml^{-1} in PBS buffer) at 4 ℃ overnight. BSA was used as a negative control. The wells were then incubated sequentially with 100 μl $well^{-1}$ of PBST plus 5% skimmed milk powder at 37 ℃ for 1h, and 100 μl $well^{-1}$ of his-tag monoclonal antibody at the indicated dilution was incubated at 37 ℃ for 1h. Horseradish perodidase (HRP) conjugated goat anti-mouse IgG diluted(1:2000) in PBST buffer was used as secondary antibody at 37 ℃ for 1 h. Five washes with PBST buffer were carried out between incubation steps. For colour development, 50 μl $well^{-1}$ of TMB mixture was added and incubated for 15 min, followed by addition of 50 μl $well^{-1}$of H_2SO_4 to stop the reaction. Absorbance was measured at 450nm in a 96-well plate reader.

Table S1: Data collection and refinement statistics.

Name	hTIM-1	hTIM-4
**Data collection**		
Resolution (Å)	50.00-1.3 (1.35-1.30)	50.00-2.30(2.38-3.30)
Unique reflections	27042 (2196)	57,162 (5,638)
Space group	*P*43212	*P*212121
Cell dimensions		
a (Å)	50.7	100.4
b (Å)	50.7	107.3
c (Å)	84.2	115.6
α (°)	90.00	90.00
β (°)	90.00	90.00
γ (°)	90.00	90.00
Redundancy	12.1(8.5)	6.5 (6.3)
Completeness (%)	97.4 (81.8)	100.0 (100.0)
R_{merge}	0.069 (0.173)	0.101 (0.617)
$I/\sigma(I)$	34.18 (12.09)	16.6 (1.8)
**Refinement**		
Resolution(Å)	1.30	2.30
No.reflections	26, 993	57, 086
R_{work}/R_{free}	0.156/0.176	0.200/0.238
No. of non-H atoms		
Protein	830	7,102
Mean B-factor(Å^2)	18.1	58.3
**Ramachandran statistics (%)**		
Most favored	95.3	96.4
Allowed	4.7	3.6
Outliers	0	0
**R.m.s.deviations**		
Bond lengths(Å)	0.013	0.009
Bond angles(°)	1.411	1.454

Values in parentheses are for highest-resolution shell.

$^aR_{merge} = \Sigma_{hkl} \Sigma_i|I(hkl)_i - \langle I(hkl)\rangle|/\Sigma_{hkl} \Sigma_i I(hkl)_i$,

$^bR_{work} = \Sigma_{hkl} |Fo(hkl) - Fc(hkl)|/\Sigma_{hkl} Fo(hkl)$.

$^cR_{free}$ was calculated for a test set of reflections (5%) omitted from the refinement.

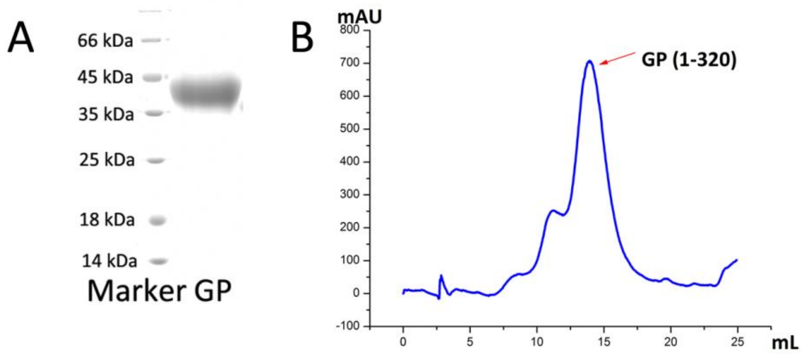

Fig. S1 Purification of EBOV GP RBD domain (1-320). (A) SDS-PAGE for EBOV GP. The molecular weight of EBOV GP from SDS-Gel indicates GP is highly glycosylation modified. (B) Size exclusion chromatography profile of EBOV GP. Eluted fractions of EBOV GP from Ni-NTA beads was concentrated and loaded on Superdex G200 size-exclusion chromatography column. Size exclusion chromatography profile of EBOV GP (absorbance at 280nm) is shown in blue.

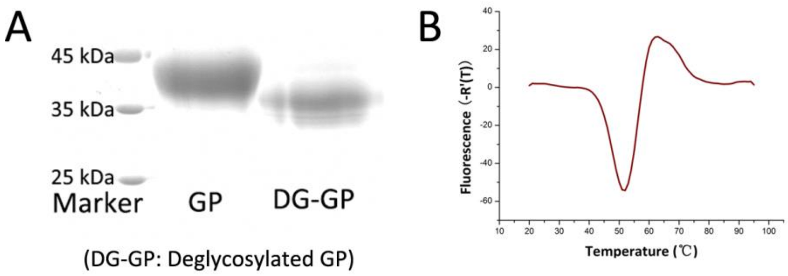

Fig. S2 Deglycosylation of EBOV GP. Purified GP was pretreated with PNGase for 1 h at 37 ℃ and then purified further by gel filtration. (A) SDS-PAGE gel shows EBOV GP and deglycosylated (DG) GP respectively. (B) Thermal stability assay suggests deglycosylated GP exhibits healthy characteristics.

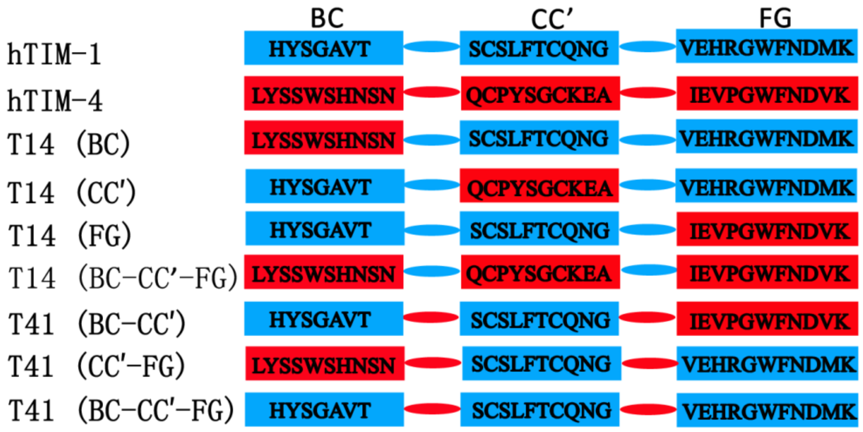

Fig. S3 Schematic diagram of chimeric hTIM-1-hTIM-4 mutants. A series of mutants were constructed by the sequential substitution of the indicated loops.

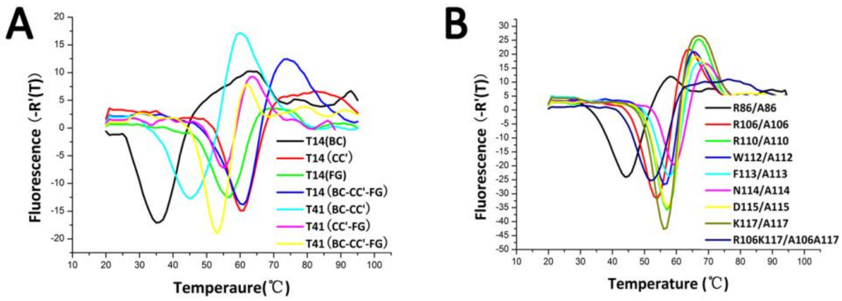

Fig. S4 Evaluation on proteins refolding successfully of the hTIM-1 mutants used in this study. (A) Chimeric hTIM-1-hTIM-4 mutants. (B) Single or double Ala substitution mutants.

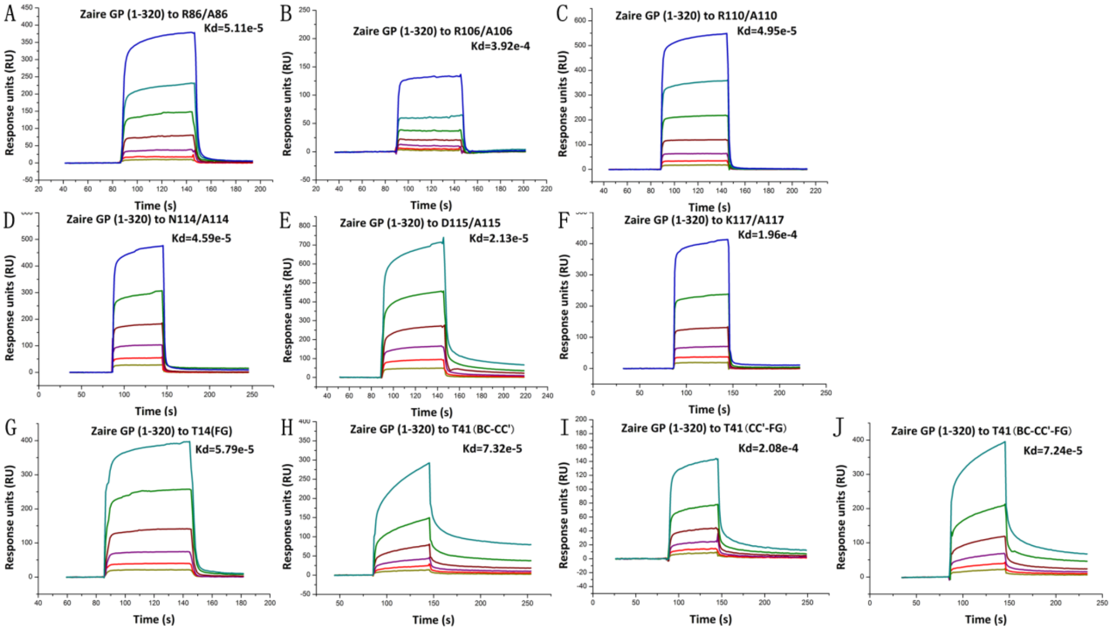

Fig. S5 Binding affinity measurements of EBOV GP to the hTIM-1 mutants. (A-F) hTIM-1 (R86/A86, R106/A106, R110/A110, N114/A114, D115/A115, and K117/A117). (G-J) T14(FG), T41(BC-CC'), T41(CC'-FG) and T41(BC-CC'-FG)

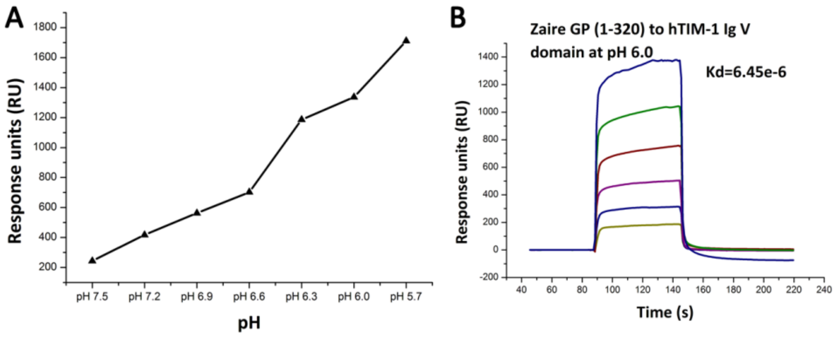

Fig. S6 Effects of pH on the binding ability of hTIM-1 to EBOV GP. (A) The interaction between hTIM-1 and EBOV GP becomes increasing as pH dropping (pH 7.5-5.7). Response units were plotted against pH series (See Materials and Methods). (B) Binding affinity measurement of hTIM-1 to EBOV GP at pH 6.0.
